# Machine learning approach to analyzing complex care coordination patterns for medically complex children

**DOI:** 10.1186/s12911-026-03691-y

**Published:** 2026-07-09

**Authors:** Saki Aoto, Yoshikazu Ito, Shintaro Morooka, Ai Ito, Akihiro Umezawa, Kohji Okamura, Tomoo Nakamura

**Affiliations:** 1 Medical Genome Center, National Center for Child Health and Development, Tokyo, Japan; 2 Department of Data Science, School of Frontier Engineering, Kitasato University, Kanagawa, Japan; 3 Center for Regenerative Medicine, National Center for Child Health and Development, Tokyo, Japan; 4 Department of General Pediatrics and Interdisciplinary Medicine, National Center for Child Health and Development Hospital, Tokyo, Japan; 5 Department of Medical AI Research, National Center for Child Health and Development, Tokyo, Japan; 6 Department of General Pediatrics and Interdisciplinary Medicine, Division of Home Care Medicine, National Center for Child Health and Development, 2-10-1 Okura, Setagaya-ku, Tokyo, 157-8535 Japan; 7 National Center for Child Health and Development, Tokyo, Japan

**Keywords:** Artificial Intelligence, Children with medical complexity, Machine learning models, Service coordination

## Abstract

**Background:**

The number of children with medical complexity (CMC) in Japan has exceeded 20,000, doubling over the past 15 years, forming a significant population group internationally. While care coordination for CMC requires consideration of growth and school attendance, the relatively small population size compared to elderly care makes it challenging for care coordinators to gain sufficient experience. We investigated whether machine learning models could assist in care coordination for CMC.

**Methods:**

We developed three tree-based gradient boosting prediction models using 1,042 care sheets from a pediatric respite facility. The first model estimated ventilator use to verify if care patterns could predict symptoms; the second predicted home doctor utilization to examine if symptoms could indicate required care; and the third estimated age to analyze school-related care needs. SHAP values were used to interpret feature importance in the prediction models. Each model was independently created and validated ten times to account for potential sampling bias among respite facility users.

**Results:**

The highest F1 scores for the three models were 0.83, 0.826, and 0.829, respectively. The ventilator model identified equipment maintenance services as highly significant; the home doctor model revealed patterns in complex service utilization; and the age model demonstrated varying important factors across school-age transitions.

**Conclusion:**

This study highlights AI’s potential in identifying crucial factors related to CMC services and its effectiveness as a valuable tool for service coordination. Despite the limited dataset of 1,042 cases, our findings emphasize the importance of digital preservation of CMC healthcare information.

**Trial registration:**

Retrospectively registered.

**Clinical trial number:**

Not applicable.

**Supplementary information:**

The online version contains supplementary material available at https://doi.org/10.1186/s12911-026-03691-y.

## Background

Children with medical complexity (CMC) are characterized by chronic conditions, functional limitations and dependence on technology, and require more health and social services than children without these conditions [1–4]. In Japan, the number of children dependent on medical technology is expected to nearly double compared to the levels from 15 years ago, with a quarter of this population on ventilators [5]. CMCs constitute a particularly important population not only in Japan, North America, and Europe but also in Asia [1–5]. Advances in medical technology have had a significant impact on the well-being of many CMCs, making quality care and services critical to their optimal health.

Caregivers face challenges in coordinating care and services due to the diversity of disease, severity and condition of CMC [6–8]. Regular hospital visits and financial constraints limit the type and amount of support available [9–11]. The number and distribution of patients and resources in the community also affect the accessibility and effectiveness of services [12].

Unlike elderly care, care coordination for CMCs with highly diverse symptoms makes it difficult for many care coordinators to gain sufficient experience, and in many cases, this burden is borne by the parents of CMCs. [13, 14]. There is a growing body of literature describing appropriate support for CMCs aimed at improving the quality of life of the children and their families, in the context of finite health care resources [9].

For older adults, AI algorithms have been used to assist in the creation of care plans [15]. However, many studies are exploring the potential of using AI to improve patient care in the area of pediatric care coordination [16], there are only a few reports on their use in service planning support for CMCs. Creating a care plan is tailored to individual patient conditions, whereas service planning support enables service providers and social workers to effectively utilize existing human resources and medical equipment resources, representing significantly different directions for AI utilization.

AI technology has greatly aided systemization in various fields, such as medicine, where data is complex and difficult to verbalize and systematize. Pediatrics is no exception. There is considerable potential for utilizing AI algorithms to capture the unmet needs of the increasing and diversifying CMCs.

Therefore, we aimed to determine the feasibility of using AI algorithms to assist in creating service plans for CMCs, and examined whether machine learning-based prediction models could:

(1) Explain the components and interactions of the comprehensive health and social services available to children with medical complexity (CMC) and their families.

(2) Identify differences in the characteristics of CMCs and the support received by different age groups.

## Methods

### Ethics approval and consent to participate

To protect patient privacy, all data was anonymized in accordance with the relevant national guidelines and regulations [17]. Specifically, this involved encoding the names of pediatric patients and their family members, removing dates from birth dates, deleting phone numbers and sub-municipal information from addresses, and changing the names of schools and facilities attended to generic institutional categories. The anonymized dataset was stored on secure, access-restricted servers, and all data transfers were encrypted using industry-standard protocols. Only authorized members of the research team had access to the data. No personally identifiable information was used in any part of the analysis or reporting. Because of the prior anonymization of the data, this is a low-risk record-based research study; as such, informed consent from the patients was not needed, as declared by the Ethical Commission at the National Center for Child Health and Development (approval reference number 2020–047). Clinical trial number: not applicable.

### Samples

In the present study, we used face sheets or care templates submitted at the time of admission to the Momiji House to collect data. Momiji House, which is a respite facility with hospice functions, was established in 2016 by the National Center for Child Health and Development (NCCHD) in Setagaya, Tokyo, Japan. This facility accepts children ages 0–18 who require intensive, high-quality medical care, including tube feeding, tracheostomy, and ventilation. The face sheets or care templates are individual information forms collected to provide CMCs with customized care. Our dataset was extracted from the care templates of patients under 18 years of age who were registered as users as of August 31, 2020. A total of 727 CMCs were eligible for inclusion, and 1042 care templates were obtained (Suppl. Table 1). The discrepancy between the number of CMCs and the number of care templates is due to the fact that patients’ requirements change significantly over time as they grow and their health status changes. Thus, more than one care template per child was obtained. What makes CMC care plans different from those for adults is that the care needs change significantly over several years due to changes in the physical condition and schooling environment as a result of growth. In other words, even for the same person, if the age of the person has changed, a completely different care plan must be proposed. Therefore, this study treated multiple care templates for the same person as independent. The care template is a handwritten form completed by the social worker or nurse in conversation with the CMC or their parents. As a result, many fields were not filled in, and some items had data points that had to be excluded.

### Study setting

The users of Momiji House are patients who receive home medical care at all times. In other words, our dataset reflects both the patient’s condition and the public medical services related to home medical care. Therefore, we constructed a ventilator model (to estimate whether the patient wears a respirator or not) as an example of estimating the patient’s condition from medical services and a visiting physician model (whether a visiting doctor is needed or not) as an example of estimating the necessary medical services from the patient’s condition. All children (including CMCs) have the right to receive an education in Japan, where education is compulsory. Therefore, by looking at the age range, it is possible to determine whether CMCs are preschoolers, elementary school students, or junior high school students and older. We divided the age range into three levels (0–5, 6–12, 13–18 years) and created a model to estimate age range based on the care requirements to determine if the nature of care changes with the educational stage (age model).

### Numeric conversion of data using a relational database

The care template data were converted into numerical values using the relational database server MariaDB 10.3.28 (https://mariadb.org/). Age, height, and weight were recorded as floating-point values in years, cm, and kg, respectively. Categorical data, such as types of schools and parents’ careers, were converted into dummy variables equal to zero or one. Frequencies, such as those of hospital visits, were calculated by dividing them by specified intervals, such as weeks, months, and years. Detail fields such as service name were assigned a value of 1 if listed, and 0 otherwise, regardless of whether anything was entered in any space. The null field was assigned to distinguish it from 0 if the required field was not filled and could not be determined by the research team through review of other available information. We examined all items, except height and weight, which were considered to be closely related to age. Finally, 366 items (education category: type of institution, frequency of commuting, means of commuting, etc.) and 16 categories (breathing, home care services, education, etc.) were considered study and predictive data set items.

### Tree-based machine learning

The care template format was revised twice during the study period. Therefore, the three formats were compared, and items where the questions and their response units were consistent were extracted as common items and selected as features to be trained and predictors (Supplementary Tables). We used the columns set as predictors and all the remaining features after removing the columns that were highly relevant to them as the training set. For example, for the prediction of Home-visiting physicians, the columns related to the frequency of physician visits were also removed from the training set.

**Fig. 1:**
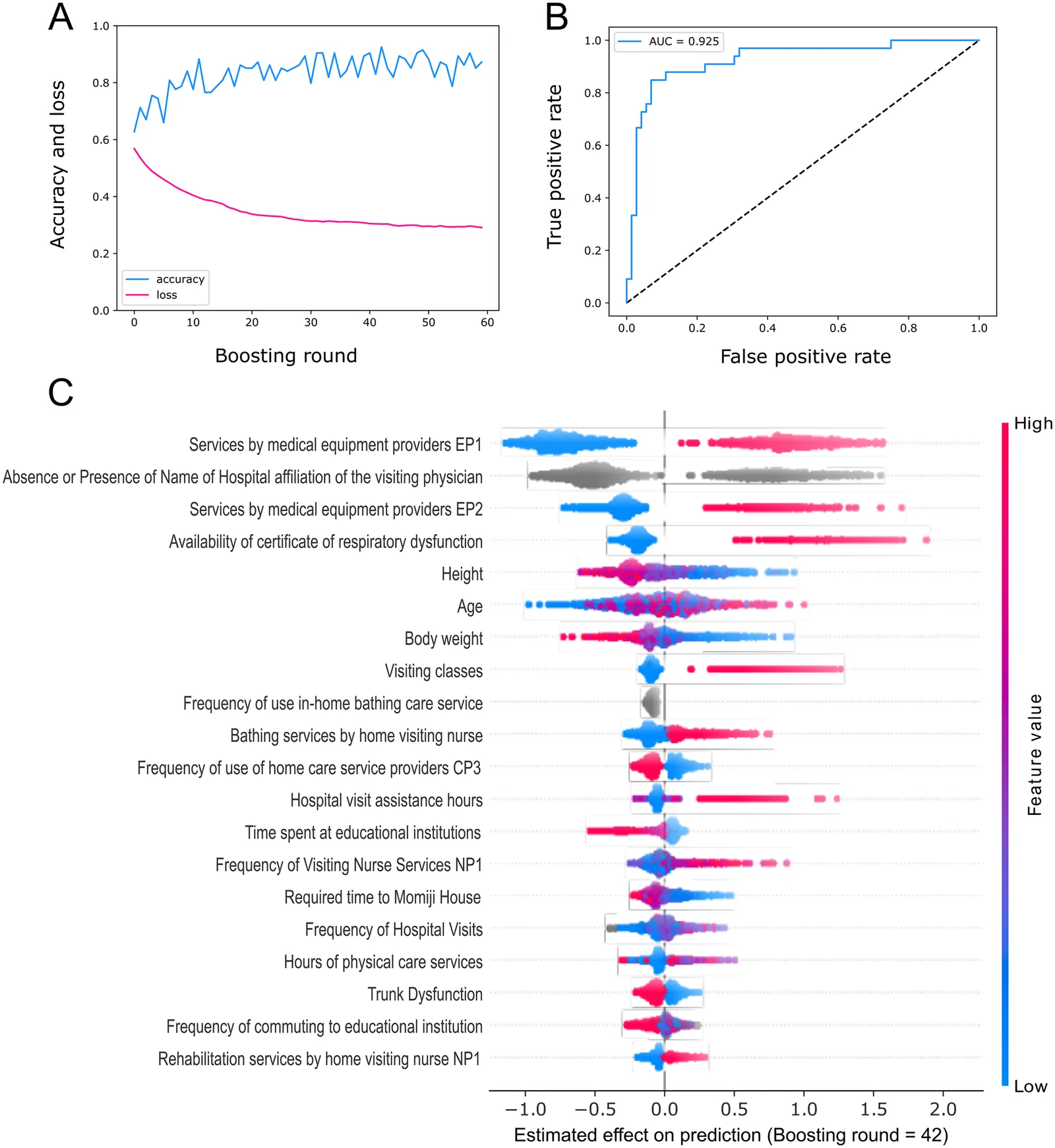
Model performance and effective features of the ventilator model. Model #3, which has the highest F1 score in 10 models, is shown in this figure. **A.** The learning curve shows validation accuracy (shown in pink) and loss score (shown in blue). **B.** ROC curves with AUC values. **C.** Feature importance plot shows the top 20 effective features on the prediction of whether CMC wear on a ventilator or not. When there are multiple entries for the same item, prefixes and numbers are assigned to distinguish them. For example, main medical equipment providers and second medical equipment providers were described as “EP1” and EP2” respectively

**Fig. 2:**
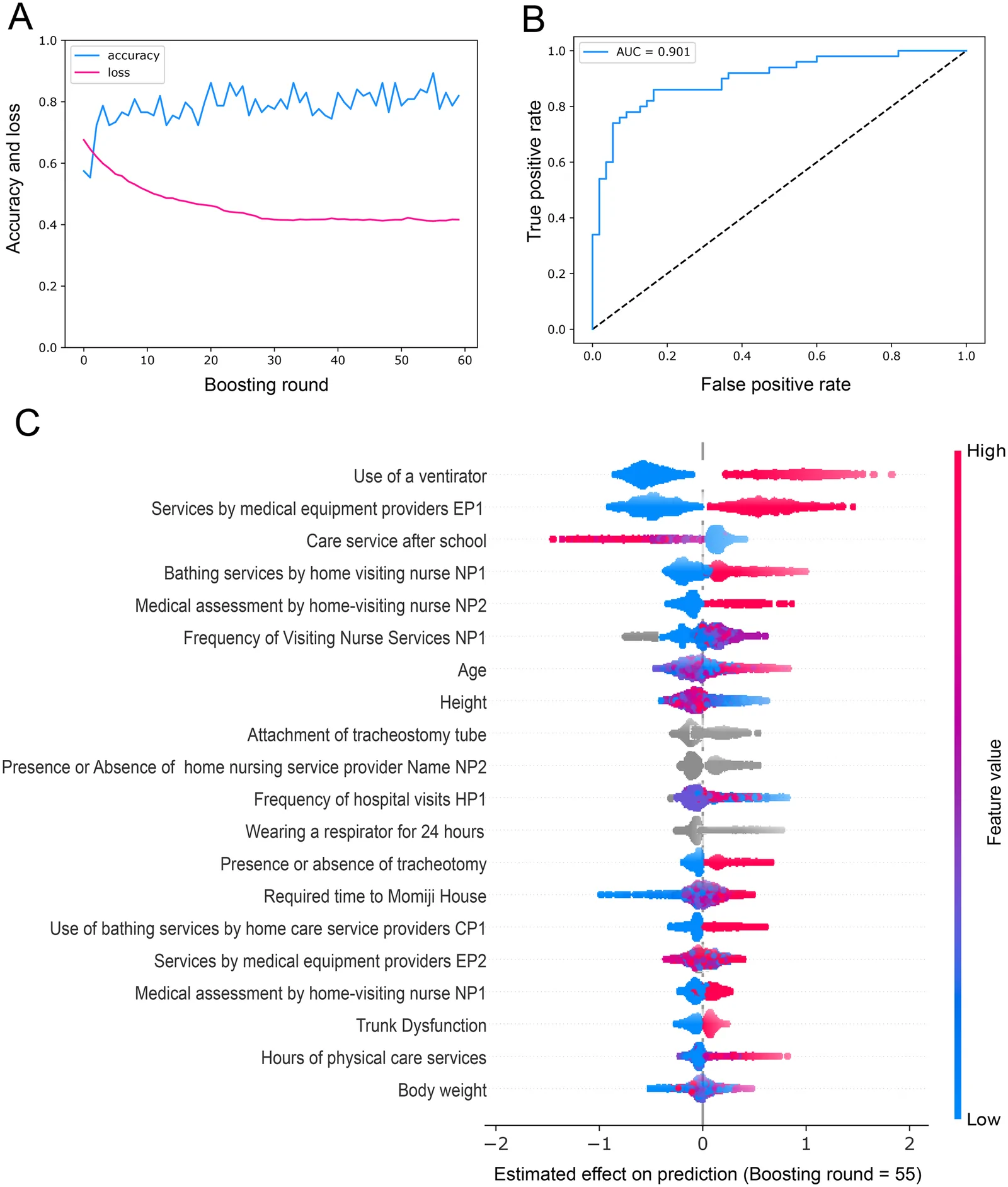
Model performance and effective features of the home-visiting physician model. Model #6, which has the highest F1 score in 10 models, is shown in this figure. **A.** The learning curve shows validation accuracy (shown in pink) and loss score (shown in blue). **B.** BROC curves with AUC value. **C.** Feature importance plot shows the top 20 effective features on the prediction whether home-visiting physician service was needed for CMC or not

**Fig. 3:**
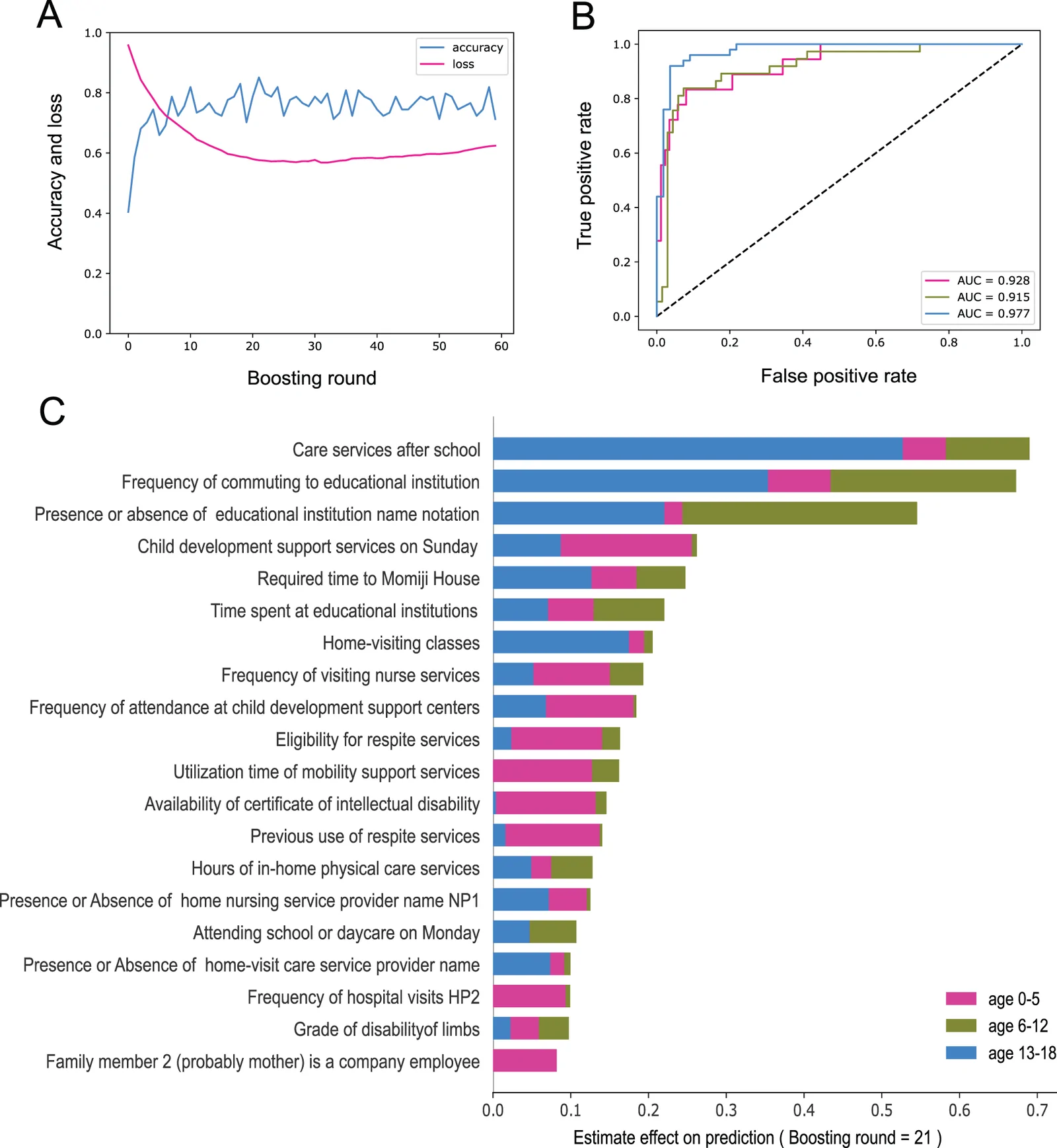
Model performance and effective features in prediction of the age model. Model #6, which has the highest F1 score in 10 models, is shown in this figure. **A.** The learning curve shows validation accuracy (pink) and loss score (blue). **B.** ROC curves with AUC values of class 0 (age 0–5, shown in pink), class 1 (age 6–12, shown in olive), and class 2 (age 13–18, shown in blue). **C.** Feature importance plot shows the top 20 effective features on age range prediction

The dataset of CMC’s physical information and care service items (1042 entries) was split into test data (105 entries, representing 10%) and pre-training data (987 entries) using Scikit-learn version 0.24.1’s train_test_split function. The pre-training data was further divided into 99 validation data entries (representing 10% in pre-training data) and 888 training data entries, with validation accuracy calculated. The training was performed using the train function of LightGBM version 3.2.1. This was chosen because LightGBM does not require feature scaling and can directly learn from data containing missing values for both numerical and categorical features. Given the diverse characteristics of our CMCs data, we determined that imputing missing values with mean values or zeros would not be advisable. Therefore, we configured the missing value parameters as use_missing = true and zero_as_missing = false. With this configuration, we intend for our model to assign branches that minimize the loss score when missing values are encountered, or to assign branches indicating “no category specified” for categorical variables. The parameters set in the LightGBM train function were {‘objective’:‘binary’, ‘metric’:‘binary_logloss’} for binary classification ventilator model and Home-visiting physician model, and {‘objective’:‘multiclass’, ‘num_class’:3} for the multiclass age model. Other hyperparameters were learned using default settings. Due to class imbalance in the CMCs data, we employed the F1 score in addition to accuracy as our primary evaluation metric. We also adopted AUC as a threshold-independent comprehensive evaluation metric for discriminative ability. As the loss score increased beyond 60 epochs, the model with the highest accuracy within 60 epochs was selected for drawing the ROC curve and calculating the F1 score using test data. Since the 1042 data entries were skewed, and F1 score was expected to vary significantly depending on the selected test data. Therefore, the process from test data creation to F1 score calculation was repeated 10 times, and the F1 score was calculated as the average of these 10 iterations. To examine the contribution of features, the Shapley additive explanation (SHAP) [16, 18–20] 0.39.0 was employed using Matplotlib 3.1.3. The performance of each model, including learning curves, ROC curves, and feature importance plots, is presented in Figs. 1, 2, 3.

We used the Hitachi HA8000/RS210 and HPE ProLiant DL360 clusters from the Center for Regenerative Medicine in NCCHD for the analyses.

## Results

Table 1 describes the characteristics of CMCs and summarizes the clinical background and types of medical care. Excluding those who did not state their sex, there were 467 boys and 407 girls. The youngest child was 4 months, the oldest was 18 years and 11 months, and the median age of the children was 7 years and 8 months. More than three-quarters of children were younger than 13 years (*N* = 833), and approximately half were aged between 6 and 13 years (*N* = 408). Most residents lived in Setagaya-ku and the neighboring city of Kawasaki. Most of the CMCs had disability certificates, and almost 80% had a level 1 (most severe) disability certificate. A total of 857 (82%) CMCs could not walk alone owing to trunk or lower-limb dysfunction. Motor dysfunction is characterized by respiratory, auditory, and circulatory dysfunctions. Most of the children used medical devices related to nutrition, such as nasogastric and gastrostomy tubes, and breathing devices, such as tracheostomies, oxygen, and ventilators; these CMCs were more common than those who used urinary catheters (Table 1). The information on the state of CMCs is complex and the relevance is difficult to determine. We therefore used LightGBM to test whether all features from face sheets (Suppl. Tables) could be predicted by other information in order to extract unknown associations. The following section presents items that were considered sufficiently predictive by machine learning the information from the CMCs.

**Table 1:** Demographic, geographic, and disability status of registrants

	N
Sex	
Male	467
Female	407
None stated	168
Age (years)	
0–2	170
3–5	255
6–12	408
13–15	124
16–18	85
Residence	
Tokyo metropolitan area	
Setagaya-ku	271
Suginami-ku	61
Ota-ku	54
Meguro-ku	44
Others	220
Suburban Tokyo	177
Tokyo surrounding area	
Kawasaki City	96
Yokohama City	32
Others	74
Other areas	14
Main affected physical region	
Trunk dysfunction	573
Upper-limb dysfunction	486
Lower-limb dysfunction	331
Upper and Lower-limb and Trunk dysfunction	401
Respiratory System	144
Auditory system	108
Others	233
Walk independently	185
Device	
Oxygen inhalation	358

HMV, home mechanical ventilation

^a^With decreasing numbers, it means severe degree of physical disability

### Ventilator model

Ventilator use is generally closely related to the severity of the child’s medical condition and is an indicator of the need for more comprehensive medical and social services. The 10 ventilator models had a mean F value of 0.75 and a mean AUC of 0.91 (Table 2). The top three features in the highest F1 score model (Fig. 1), “Services by medical equipment providers EP1,” “Services by medical equipment providers EP2,” “Absence or Presence of Name of Hospital affiliation of the visiting physician” are identical for the 10 models, and these indicate the contact information for medical equipment failure/repair and the visiting physician. Other items that were not high on the list but were common across the 10 models were “Height” and “Age,” which indicate physical characteristics, “Visiting classes” and “Frequency of commuting to educational institution,” which are related to education, and “Home care services”, “Availability of certificate of respiratory dysfunction”, “Frequency of use in-home bathing care service”, “Bathing services by home visiting nurse”, which is related to in-home services.

**Table 2:** F1 scores in 10 learning models

	model #										average
	1	2	3	4	5	6	7	8	9	10	
Ventirator model	0.814815	0.6875	**0.830769**	0.76	0.724138	0.708333	0.677966	0.818182	0.813559	0.666667	0.7501929
Home-visiting physician model	**0.826087**	0.767442	0.764045	0.717949	0.756098	0.735632	0.717949	0.64	0.705882	0.75	0.7381084
Age model (macroF1)	0.710787	0.745444	0.705927	0.808387	0.758653	**0.829665**	0.760587	0.785672	0.785305	0.788543	0.767897
Age model (Label 0: age 0–5)	0.615385	0.634146	0.702703	0.702703	0.666667	0.75	0.648649	0.722222	0.73913	0.76	0.6941605
Age model (Label 1: age 6–12)	0.698795	0.704225	0.591549	0.787879	0.739726	0.833333	0.722222	0.741935	0.707692	0.698413	0.7225769
Age model (Label 2 : age 13–18)	0.818182	0.897959	0.823529	0.934579	0.869565	0.90566	0.910891	0.892857	0.909091	0.907216	0.8869529

The F1 score was calculated using learning models independently created 10 times, taking into account the bias in the test data. Since the age model is a multiclass classification, the macro F1 score was employed instead of the F1score in the binary classification

### Home-visiting physician model

Home-visiting physician services are more susceptible to local government welfare policies compared to other medical services because they require cooperation with local clinics. Therefore, whether a patient receives this service should serve as an indicator of both their more complex health status and their high accessibility to other services in their local environment. The mean F value of the 10 home-visiting physician models was 0.738, and the mean AUC was 0.869 (Table 2). In ranking the contribution of the features, “Use of a ventilator” and “Services by medical equipment providers EP1” were the top two features common to the 10 models and were found to be important for prediction (Fig. 2). Other features common to the 10 models that positively contributed to home-visiting physician use were “Bathing services by home visiting nurse NP1,” “Medical assessment by home-visiting nurse NP2,” “Age” and ‘Required time to Momiji House”, and the negative contributors were ‘Care service after school’ and ‘Height’.

### Age model

Age is the most critical factor in determining which services are available to CMCs, as growing up necessitates transitions from childcare facilities to educational institutions and requires upsizing medical equipment. The purpose of this model is to estimate the age range and highlight services that require modifications to the care plan according to growth. The average macro F1 value of 10 independently created Age models was 0.768 (Table 2). In Fig. 3, the ROC curve and feature contribution by SHAP for the model with the highest macro F1 value are presented, and the AUC for each age category reached over 0.85, with a particularly high AUC exceeding 0.95 for predictions in the 13–18 age group. The top three items remain identical. In age estimation, after-school day services contributed most significantly, particularly in identifying middle and high school students. In the “Presence or absence of educational institution name notation” item, this field was often filled when Momiji House was considering coordination, especially for special support schools. On the other hand, items notably contributing to estimating preschool children included Child development support services on Sunday, mobility support, intellectual disability, and respite services. An interesting item that was ranked 20th was “occupation of family member 2”; 71 of the 82 CMCs with “family member 2” as “company employee” were under the age of 5.

All presented models achieved F1 scores exceeding 0.7, which indicates robust performance given that values above 0.6 are considered practically viable. Additionally, all models demonstrated excellent discriminative ability with AUC scores above 0.9.

## Discussion

In the ventilator model, “Availability of certificate of respiratory dysfunction” is an item that should be directly related to the inferential results of the ventilator model, but the more substantial items, ‘name of respiratory repair service company’ and ‘visiting nurse,’ had higher contributions. Overall, the ventilator model was positively correlated with services related to education and medical equipment maintenance in addition to home-based services. In Japan, medical equipment maintenance is bundled with the acquisition of medical devices and is therefore not a subject for planning; however, there are multiple services available for receiving education. In other words, our model demonstrated the importance of proposing education-related services, including visiting classes, for CMCs who are determined to need or are using ventilators.

The home-visiting physician model showed that CMCs who use life-sustaining equipment, mainly ventilators, tend to use visiting physicians. Although the direction of the relationship was unclear in the ventilator use model, it predicted a positive relationship between home-visiting physicians and ventilator users. As expected, home physician services were positively correlated with complex and multiple use of other nursing and care services. The positive contribution of “Age,” “Required time to Momiji House”, and the negative contribution of “Care service after school” and “Height” evoked a clinical picture of poor physical development for age, mobility problems, and few educational and childcare opportunities. Our model was able to link CMC with health conditions that require them to spend much of the day lying in bed to the use of visiting physician services. These results indicate that our model identifies CMCs who are utilizing or have been recognized as needing home visiting physician services as requiring comprehensive home-based service support by nurses frequently and regularly. Furthermore, it suggests that care coordinators or AI care plan support need to regularly monitor whether such support reaches the appropriate level that should be provided by local governments and communities.

The ventilator model and home-visiting physician model showed remarkable similarity, with both revealing profiles of individuals with limited mobility who relied heavily on home-based services. This similarity is likely attributable to the fact that many users of home visiting physician services were ventilator dependent. However, these two models differed dramatically in terms of education. While the Ventilator Model ranked services related to transportation to multiple educational institutions among the top 20 features, the home-visiting physician model showed negative correlations with after-school day service utilization. This suggests that despite both models targeting CMCs with respiratory impairments, they represent CMCs at different educational levels. AI care planning support that combines these two models would be able to propose care plans that prioritize education or place greater emphasis on health status according to individual CMCs.

The Age model also revealed that after-school day services are more actively utilized by middle and high school students compared to elementary school students. In our country, facilities specialized for disabled babies and infants in daycare settings are extremely rare, and the top items were helpful in estimating school-age children. However, items contributing to estimating preschool children did not correspond to pre-school CMCs and actually had a negative effect on determining pre-school status. From these findings, our model can distinguish between age groups requiring childcare, primary education, secondary education, or higher, confirming that each group requires different services. In other words, our model is capable of proposing age-appropriate care services.

Another notable finding from the Age model was regarding ‘occupation of family number 2’ which ranked 20th. In Japan, it is customary to write the names of family members in the order of father and mother, so “occupation of family member 2” can be translated as the mother’s occupation. Although it is difficult to estimate the positive or negative effect of each item in the multiclass classification, the finding that 71 of 82 CMCs with “family member 2” as “company employee” were under the age of 5 suggests that the employment rate of parent 2 decreases when the child reaches school age. This may indicate the need to support the employment of caregivers after the CMC’s enrollment in school. In Japan, there is a problem called the “first-grade wall,” where even mothers of healthy children have no place to leave their children after school and are forced to quit their jobs. Additionally, elementary schools often lack adequate support systems, requiring parents to accompany their children to school for extended periods, making it difficult for parents to maintain their employment.

A major difference between CMC care and care for elderly or adult populations is the need to adapt to child development and related environmental changes, requiring frequent revisions of care plans for CMCs within short time periods. The advantage of AI algorithms is that once a model is established, the time and cost required for prediction execution are minimal. AI algorithms that operate automatically and regularly, proposing age-appropriate childcare and education-related services or support services for family employment of CMCs, would provide substantial assistance to families of CMCs.

These results indicate that our models maintain high predictive accuracy across various threshold configurations, enabling flexible deployment that can expand care service coverage according to regional healthcare resource constraints. Our retrospective analysis of predictive models demonstrated that AI technology in conjunction with MLPM can identify key factors provided to CMCs and their families, as well as uncover relationships between these factors. Additionally, this study revealed differences in CMC characteristics and the support received across various age groups.

This study had several limitations. First, the data in the template was designed for appropriate medical care and non-care coordination. Therefore, insufficient information is available regarding codification. Second, this study included only data from patients from a single facility. The model used in the present study may not perform as well when applied broadly to children receiving medical care in Japan, as the majority of users of Momiji House are children with more severely ill conditions who use the national children’s hospital, which actively promotes cooperation with visiting doctors. Finally, in this study, patient information was obtained from hand-written face sheets, which are the most common recording methods used in respite facilities in Japan. As such, there were many variations in data quality and completeness. LightGBM is a learning algorithm that tolerates null entries, but whether an entry is incomplete in the sense of “no” or not in the sense of “null” responses has a significant impact on prediction. As described above, it became clear that there are limitations in capturing the overall picture of CMCs in our country using conventional handwritten face sheets. If a digital CMC registry with the same format that does not allow for incompleteness can be operated, it would become easier to combine patient data from other facilities, allowing us to make our model more generalizable.

Nijman et al. described that AI has the power to revolutionize pediatric health care but currently fails to reach the clinical workflow and that a strategy toward AI with clinical impact may lead to higher-quality, reliable, and transparent AI models fit for the problem [21]. To build a systematic support system for CMCs, it is essential to accumulate digitized data based on a common national format that conforms to the International Classification of Functioning, Disability, and Health and other standards [22, 23]. Once such a framework is in place, machine learning-based models, which consist of deep-learning and tree-based algorithms, will help provide recommendations on policy and public service delivery as well as coding that is more in line with the actual conditions of patients and their families.

## Conclusion

This study demonstrated that machine learning-based predictive models using LightGBM can identify key relationships between CMC characteristics and the services they receive, with all three models achieving F1 scores exceeding 0.7 and AUC scores above 0.9. This study indicates the potential of using AI technology in conjunction with machine learning based prediction models to assist healthcare professionals in treating CMCs based on the creation of service plans. Integrating each model to create a comprehensive AI care plan service is a realistic and achievable goal. In Japan, remarkable advances in neonatal and pediatric medicine have led to an increase in the number of CMCs, while the proportion of severe CMCs has decreased. Our public services for CMCs have traditionally targeted bedridden CMCs. If we can conduct multi-center collaborative research using a digital CMC registry and generalize the model, we believe it would lead to proposals for new services targeting mild CMCs and their families, such as career support for CMC’s mothers. To utilize the assistance of AI effectively requires a large number of samples and the preparation of a database of meticulously designed and compiled data. Establishing a national digital database for CMCs will be a critical next step toward realizing AI-assisted care planning that benefits both CMCs and their families.

## Electronic supplementary material

Below is the link to the electronic supplementary material.

Supplementary Material 1

Supplementary Material 2. Supplemental Figure 1. Top 20 feature importance plot of ventilator model #1~10. The color bar shows feature values (low value shown in blue to high value shown in pink). The horizontal axis shows the direction of the effects of features. Supplemental Figure 2. Top 20 feature importance plot of home-visiting physician model #1~10. The color bar shows feature values (low value shown in blue to high value shown in pink). The horizontal axis shows the direction of the effects of features. Supplemental Figure 3. Top 20 feature importance plot of age model #1~10. The horizontal axis shows the magnitude of the effects of features. Class 0, Class 1, and Class 2 show CMC with ages 0-5, 6-12, and 13-18, respectively

## Data Availability

All data analyzed during this study are included in this published article and its supplementary information files. The datasets generated during the current study are available from the corresponding author upon reasonable request.

## Abbreviations

CMC: Children with medical complexity
AI: Artificial Intelligence
LightGBM: Light Gradient Boosting Machine
SHAP: SHapley Additive exPlanations
AUC: Area Under the Curve
ROC: Receiver Operating Characteristic
MLPM: Multi-Layer Perceptron Model
