# Supplementary material for: Machine learning approach to analyzing complex care coordination patterns for medically complex children
Source: BMC Med Inform Decis Mak. 2026 Jul 9;26:385. doi: 10.1186/s12911-026-03691-y (PMC13632045; doi:10.1186/s12911-026-03691-y)

Figure 1

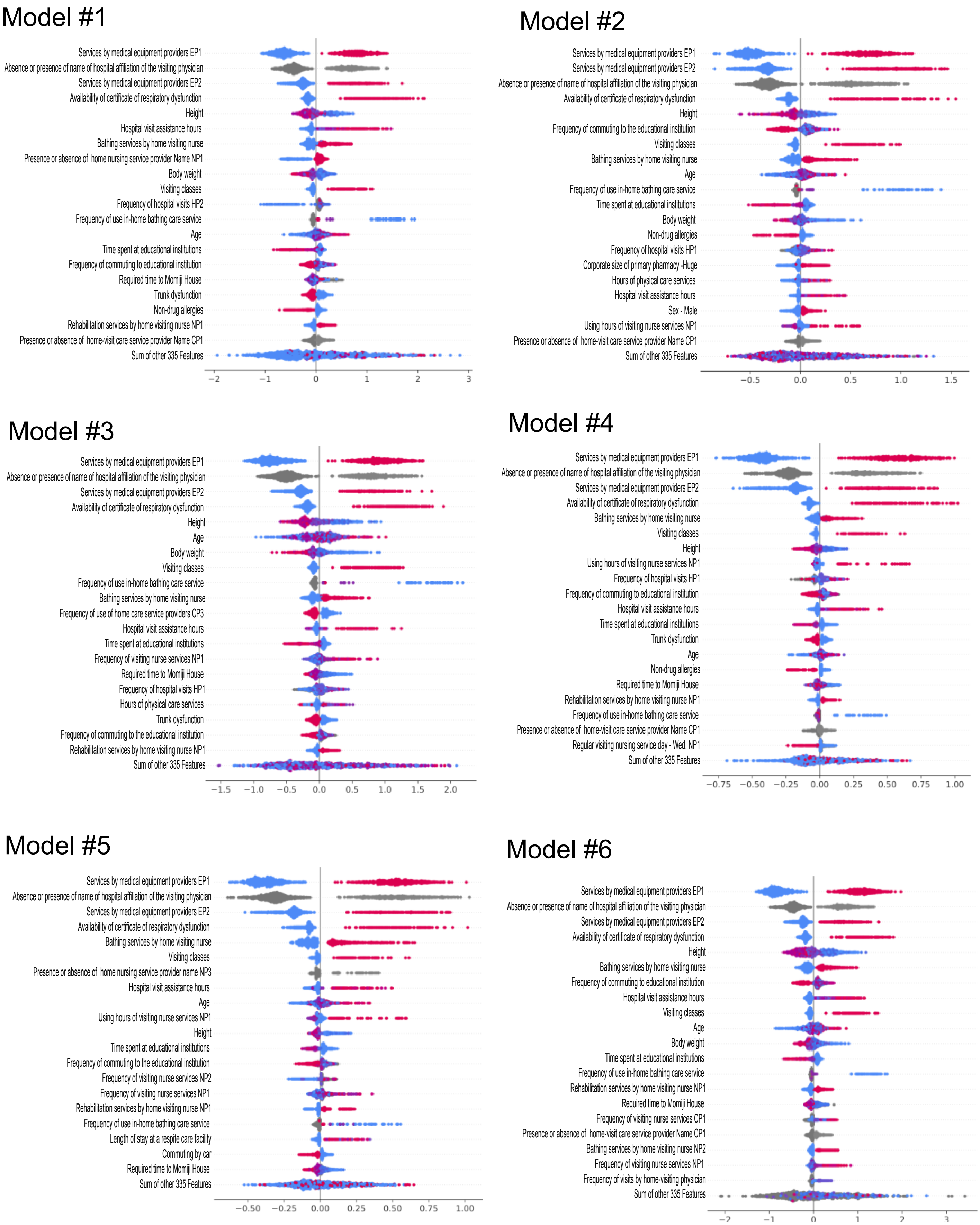
Model #1
Services by medical equipment providers EP1
Absence or presence of name of hospital affiliation of the visiting physician
Services by medical equipment providers EP2
Availability of certificate of respiratory dysfunction
Height
Hospital visit assistance hours
Bathing services by home visiting nurse
Presence or absence of home nursing service provider Name NP1
Body weight
Visiting classes
Frequency of hospital visits HP2
Frequency of use in-home bathing care service
Age
Time spent at educational institutions
Frequency of commuting to educational institution
Required time to Momiji House
Trunk dysfunction
Non-drug allergies
Rehabilitation services by home visiting nurse NP1
Presence or absence of home-visit care service provider Name CP1
Sum of other 335 Features
−2
−1
0
1
2
3
Model #2
Services by medical equipment providers EP1
Services by medical equipment providers EP2
Absence or presence of name of hospital affiliation of the visiting physician
Availability of certificate of respiratory dysfunction
Height
Frequency of commuting to the educational institution
Visiting classes
Bathing services by home visiting nurse
Age
Frequency of use in-home bathing care service
Time spent at educational institutions
Body weight
Non-drug allergies
Frequency of hospital visits HP1
Corporate size of primary pharmacy -Huge
Hours of physical care services
Hospital visit assistance hours
Sex - Male
Using hours of visiting nurse services NP1
Presence or absence of home-visit care service provider Name CP1
Sum of other 335 Features
−0.5
0.0
0.5
1.0
1.5
Model #3
Services by medical equipment providers EP1
Absence or presence of name of hospital affiliation of the visiting physician
Services by medical equipment providers EP2
Availability of certificate of respiratory dysfunction
Height
Age
Body weight
Visiting classes
Frequency of use in-home bathing care service
Bathing services by home visiting nurse
Frequency of use of home care service providers CP3
Hospital visit assistance hours
Time spent at educational institutions
Frequency of visiting nurse services NP1
Required time to Momiji House
Frequency of hospital visits HP1
Hours of physical care services
Trunk dysfunction
Frequency of commuting to the educational institution
Rehabilitation services by home visiting nurse NP1
Sum of other 335 Features
−1.5
−1.0
−0.5
0.0
0.5
1.0
1.5
2.0
Model #4
Services by medical equipment providers EP1
Absence or presence of name of hospital affiliation of the visiting physician
Services by medical equipment providers EP2
Availability of certificate of respiratory dysfunction
Bathing services by home visiting nurse
Visiting classes
Height
Using hours of visiting nurse services NP1
Frequency of hospital visits HP1
Frequency of commuting to educational institution
Hospital visit assistance hours
Time spent at educational institutions
Trunk dysfunction
Age
Non-drug allergies
Required time to Momiji House
Rehabilitation services by home visiting nurse NP1
Frequency of use in-home bathing care service
Presence or absence of home-visit care service provider Name CP1
Regular visiting nursing service day - Wed. NP1
Sum of other 335 Features
−0.75
−0.50
−0.25
0.00
0.25
0.50
0.75
1.00
Model #5
Services by medical equipment providers EP1
Absence or presence of name of hospital affiliation of the visiting physician
Services by medical equipment providers EP2
Availability of certificate of respiratory dysfunction
Bathing services by home visiting nurse
Visiting classes
Presence or absence of home nursing service provider name NP3
Hospital visit assistance hours
Age
Using hours of visiting nurse services NP1
Height
Time spent at educational institutions
Frequency of commuting to the educational institution
Frequency of visiting nurse services NP2
Frequency of visiting nurse services NP1
Rehabilitation services by home visiting nurse NP1
Frequency of use in-home bathing care service
Length of stay at a respite care facility
Commuting by car
Required time to Momiji House
Sum of other 335 Features
−0.50
−0.25
0.00
0.25
0.50
0.75
1.00
Model #6
Services by medical equipment providers EP1
Absence or presence of name of hospital affiliation of the visiting physician
Services by medical equipment providers EP2
Availability of certificate of respiratory dysfunction
Height
Bathing services by home visiting nurse
Frequency of commuting to educational institution
Hospital visit assistance hours
Visiting classes
Age
Body weight
Time spent at educational institutions
Frequency of use in-home bathing care service
Rehabilitation services by home visiting nurse NP1
Required time to Momiji House
Frequency of visiting nurse services CP1
Presence or absence of home-visit care service provider Name CP1
Bathing services by home visiting nurse NP2
Frequency of visiting nurse services NP1
Frequency of visits by home-visiting physician
Sum of other 335 Features
−2
−1
0
1
2
3

Model #7

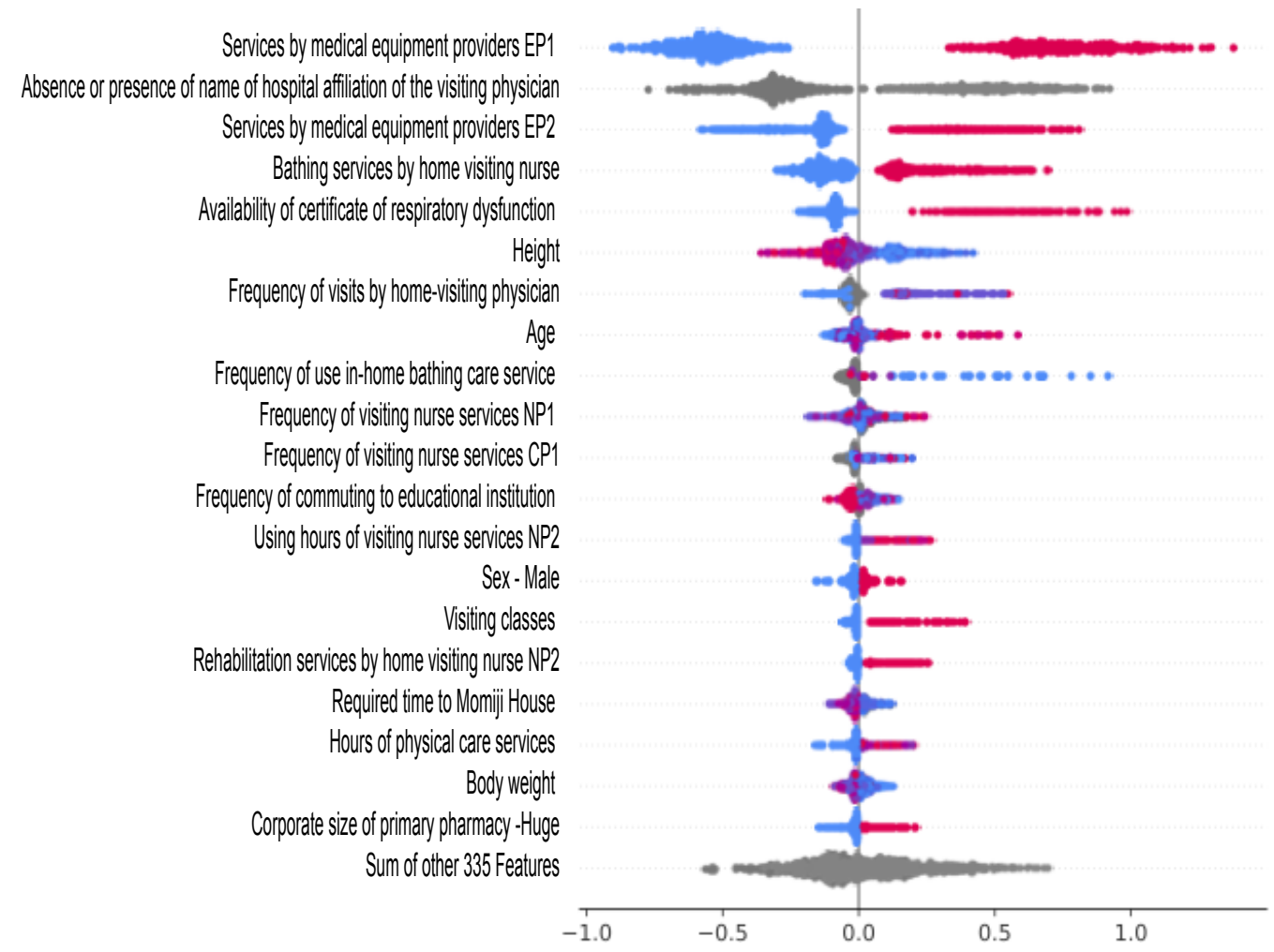

Model #8

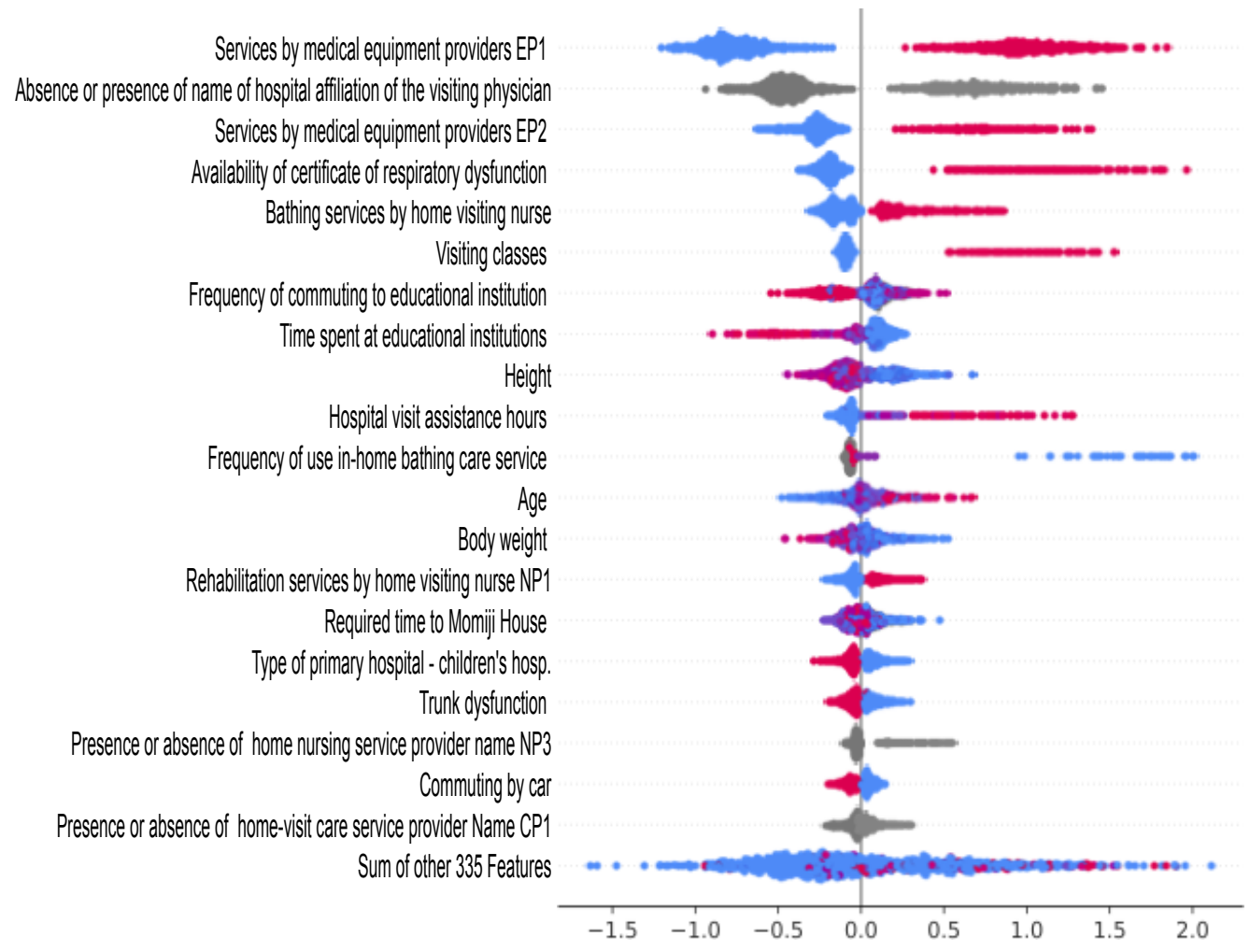

Model #9

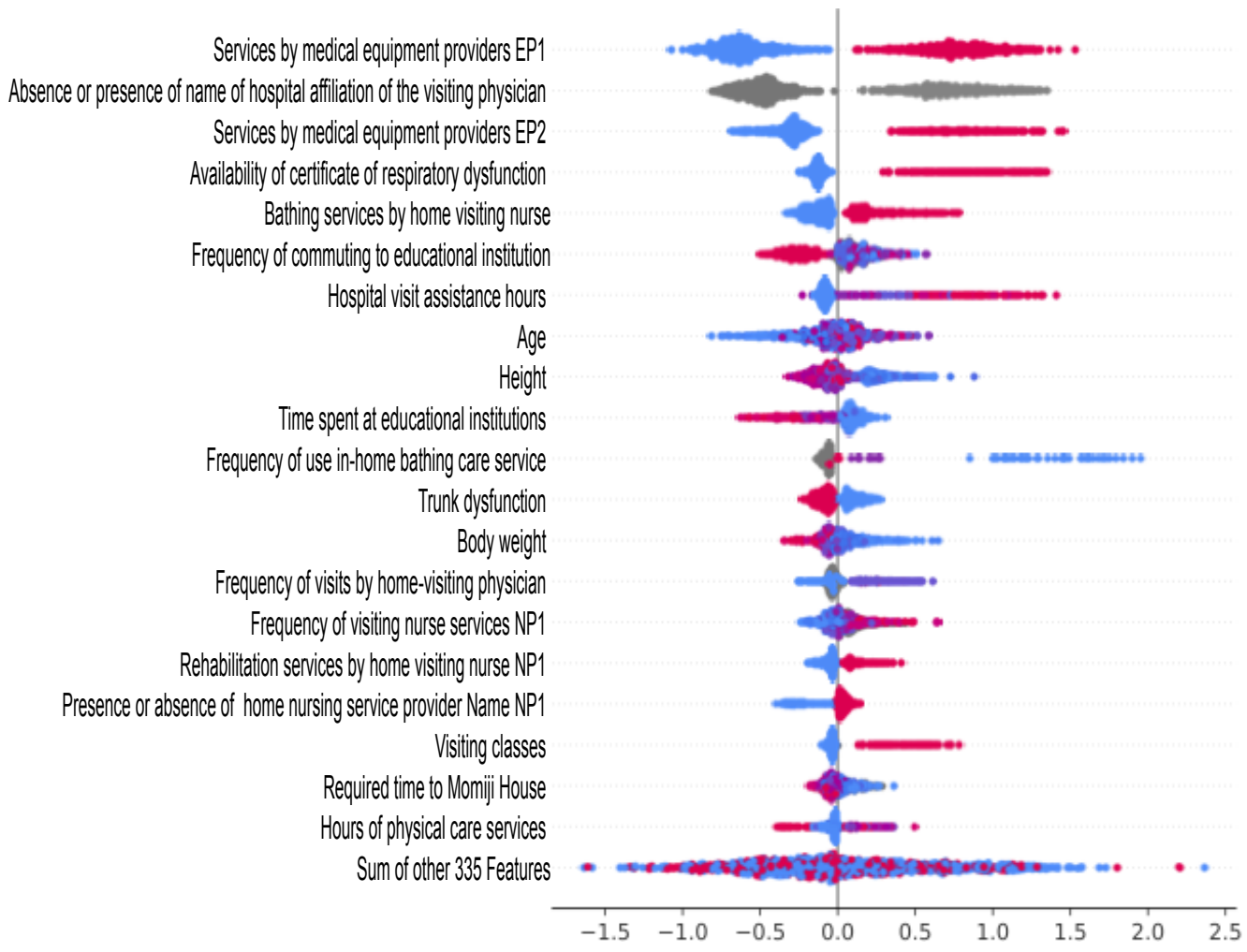

Model #10

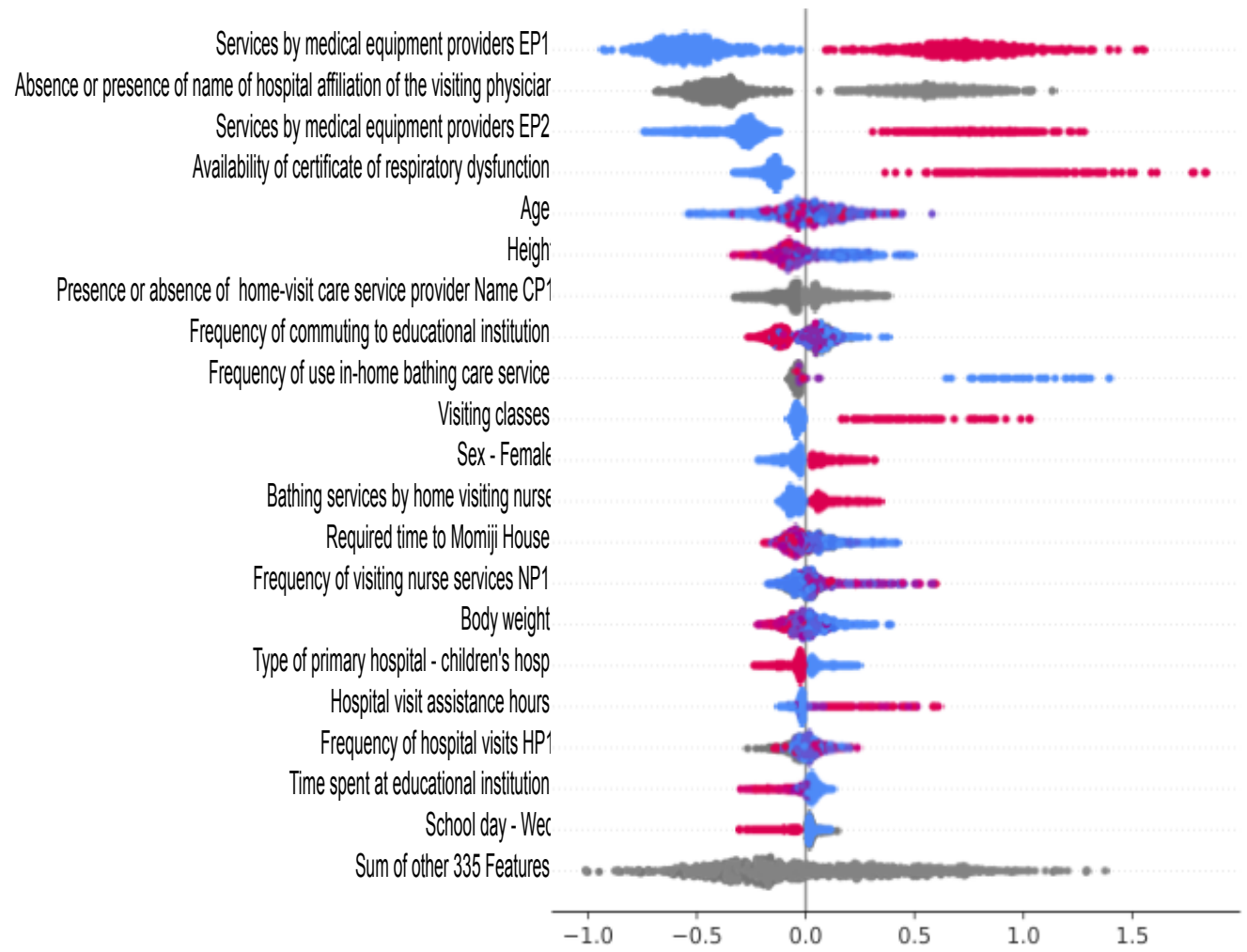

Figure 2

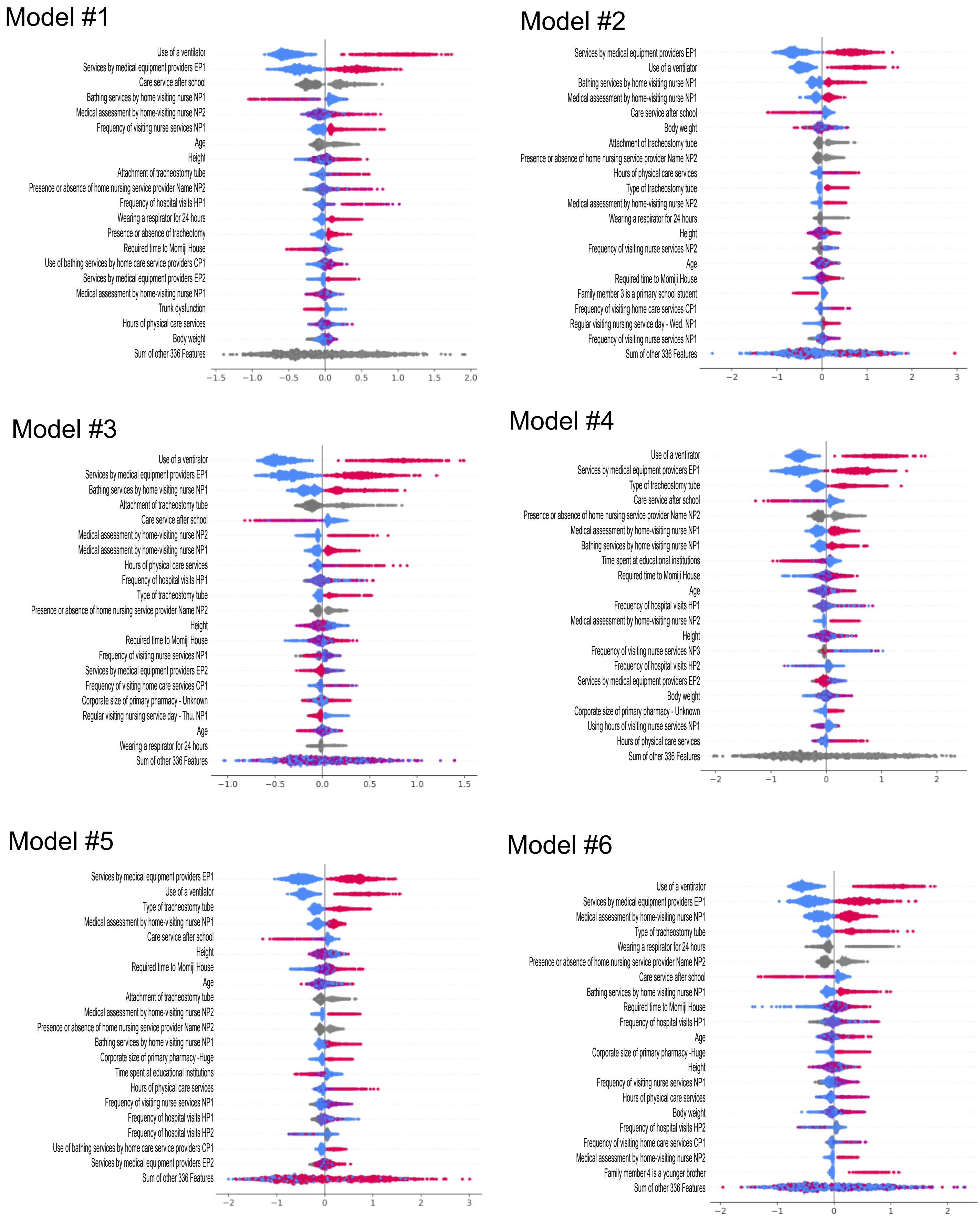
Model #1
Use of a ventilator
Services by medical equipment providers EP1
Care service after school
Bathing services by home visiting nurse NP1
Medical assessment by home-visiting nurse NP2
Frequency of visiting nurse services NP1
Age
Height
Attachment of tracheostomy tube
Presence or absence of home nursing service provider Name NP2
Frequency of hospital visits HP1
Wearing a respirator for 24 hours
Presence or absence of tracheotomy
Required time to Momiji House
Use of bathing services by home care service providers CP1
Services by medical equipment providers EP2
Medical assessment by home-visiting nurse NP1
Trunk dysfunction
Hours of physical care services
Body weight
Sum of other 336 Features
−1.5 −1.0 −0.5 0.0 0.5 1.0 1.5 2.0
Model #2
Services by medical equipment providers EP1
Use of a ventilator
Bathing services by home visiting nurse NP1
Medical assessment by home-visiting nurse NP1
Care service after school
Body weight
Attachment of tracheostomy tube
Presence or absence of home nursing service provider Name NP2
Hours of physical care services
Type of tracheostomy tube
Medical assessment by home-visiting nurse NP2
Wearing a respirator for 24 hours
Height
Frequency of visiting nurse services NP2
Age
Required time to Momiji House
Family member 3 is a primary school student
Frequency of visiting home care services CP1
Regular visiting nursing service day - Wed. NP1
Frequency of visiting nurse services NP1
Sum of other 336 Features
−2 −1 0 1 2 3
Model #3
Use of a ventirator
Services by medical equipment providers EP1
Bathing services by home visiting nurse NP1
Attachment of tracheostomy tube
Care service after school
Medical assessment by home-visiting nurse NP2
Medical assessment by home-visiting nurse NP1
Hours of physical care services
Frequency of hospital visits HP1
Type of tracheostomy tube
Presence or absence of home nursing service provider Name NP2
Height
Required time to Momiji House
Frequency of visiting nurse services NP1
Services by medical equipment providers EP2
Frequency of visiting home care services CP1
Corporate size of primary pharmacy - Unknown
Regular visiting nursing service day - Thu. NP1
Age
Wearing a respirator for 24 hours
Sum of other 336 Features
−1.0 −0.5 0.0 0.5 1.0 1.5
Model #4
Use of a ventirator
Services by medical equipment providers EP1
Type of tracheostomy tube
Care service after school
Presence or absence of home nursing service provider Name NP2
Medical assessment by home-visiting nurse NP1
Bathing services by home visiting nurse NP1
Time spent at educational institutions
Required time to Momiji House
Age
Frequency of hospital visits HP1
Medical assessment by home-visiting nurse NP2
Height
Frequency of visiting nurse services NP3
Frequency of hospital visits HP2
Services by medical equipment providers EP2
Body weight
Corporate size of primary pharmacy - Unknown
Using hours of visiting nurse services NP1
Hours of physical care services
Sum of other 336 Features
−2 −1 0 1 2
Model #5
Services by medical equipment providers EP1
Use of a ventilator
Type of tracheostomy tube
Medical assessment by home-visiting nurse NP1
Care service after school
Height
Required time to Momiji House
Age
Attachment of tracheostomy tube
Medical assessment by home-visiting nurse NP2
Presence or absence of home nursing service provider Name NP2
Bathing services by home visiting nurse NP1
Corporate size of primary pharmacy -Huge
Time spent at educational institutions
Hours of physical care services
Frequency of visiting nurse services NP1
Frequency of hospital visits HP1
Frequency of hospital visits HP2
Use of bathing services by home care service providers CP1
Services by medical equipment providers EP2
Sum of other 336 Features
−2 −1 0 1 2 3
Model #6
Use of a ventirator
Services by medical equipment providers EP1
Medical assessment by home-visiting nurse NP1
Type of tracheostomy tube
Wearing a respirator for 24 hours
Presence or absence of home nursing service provider Name NP2
Care service after school
Bathing services by home visiting nurse NP1
Required time to Momiji House
Frequency of hospital visits HP1
Age
Corporate size of primary pharmacy -Huge
Height
Frequency of visiting nurse services NP1
Hours of physical care services
Body weight
Frequency of hospital visits HP2
Frequency of visiting home care services CP1
Medical assessment by home-visiting nurse NP2
Family member 4 is a younger brother
Sum of other 336 Features
−2 −1 0 1 2

Model #7

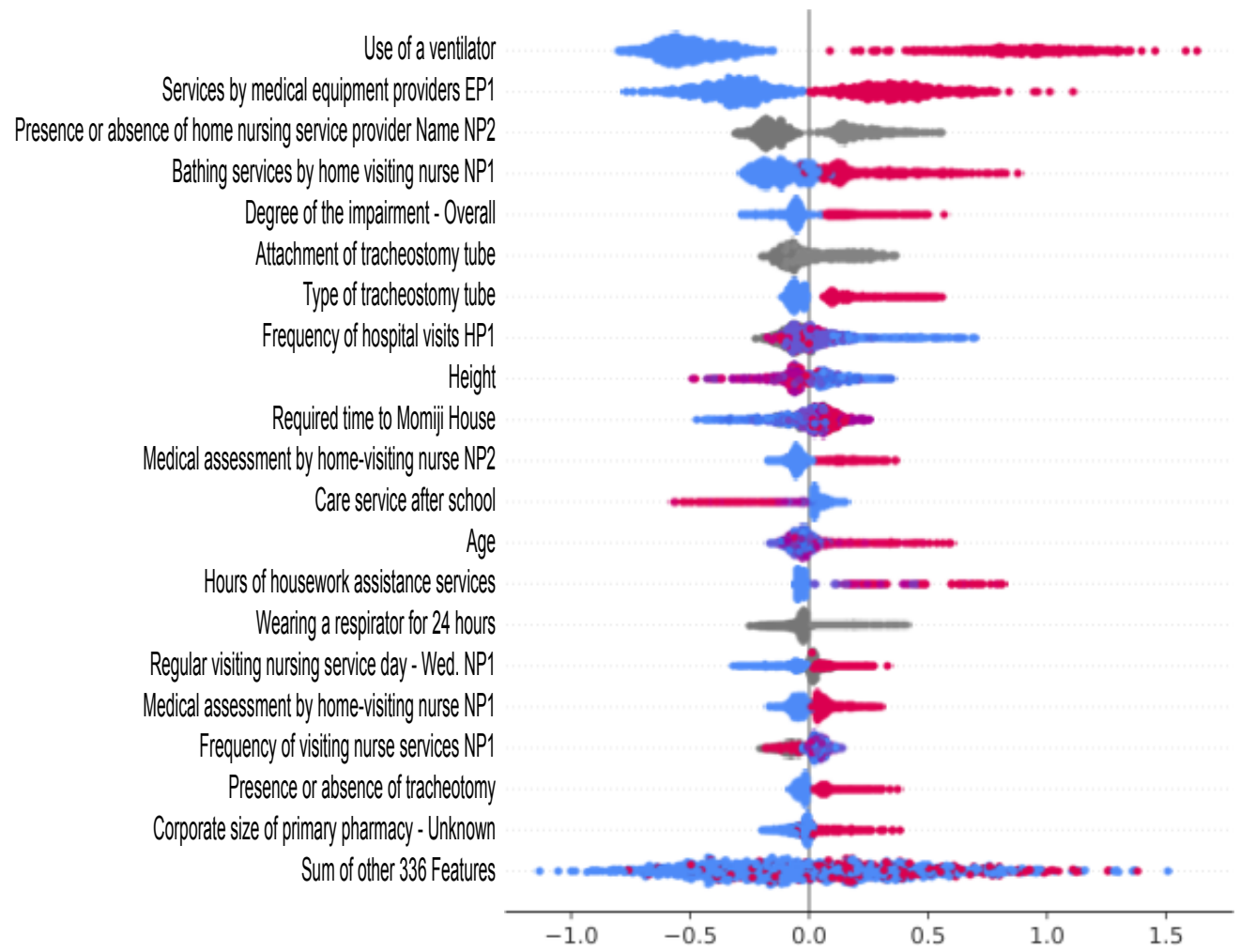

Model #8

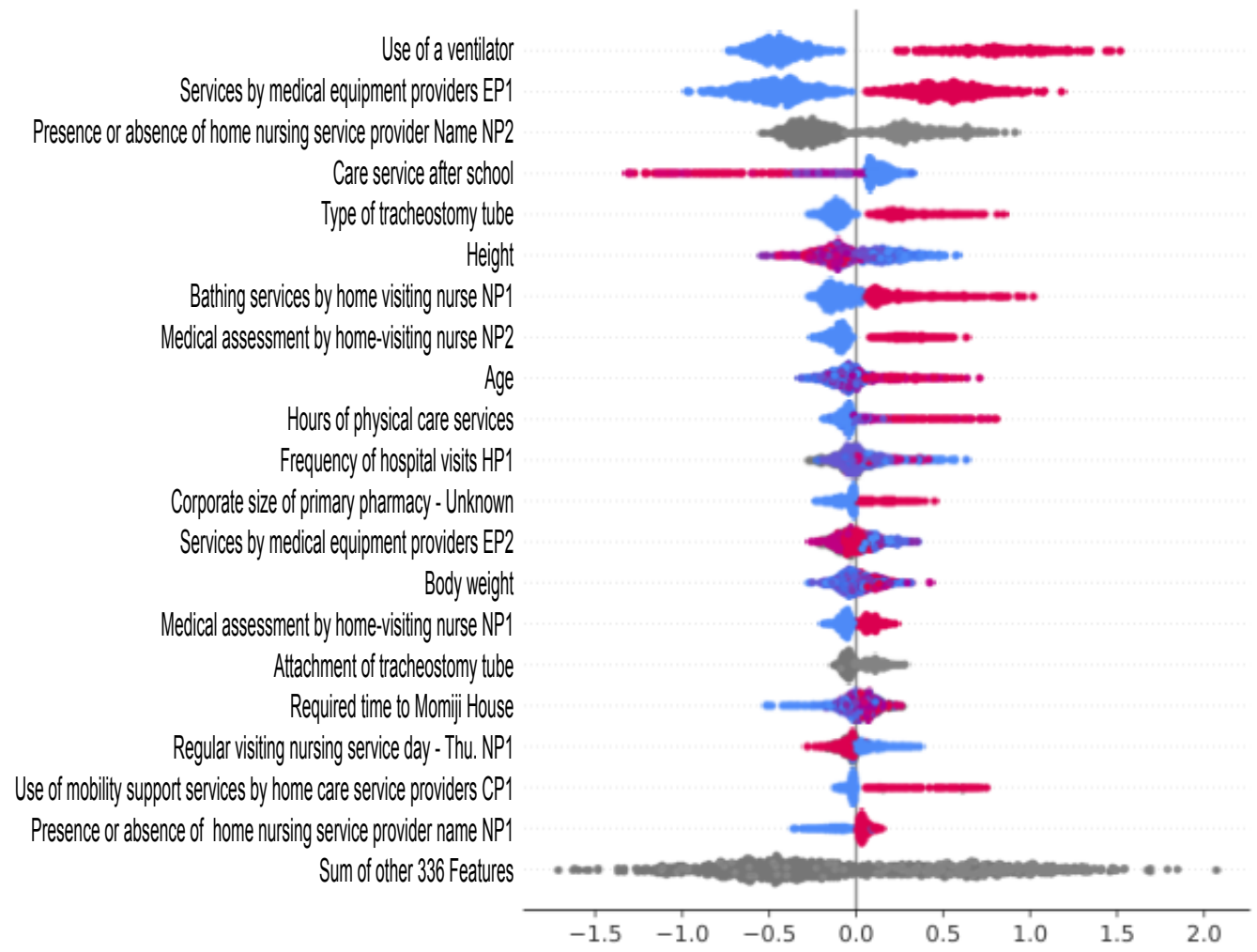

Model #9

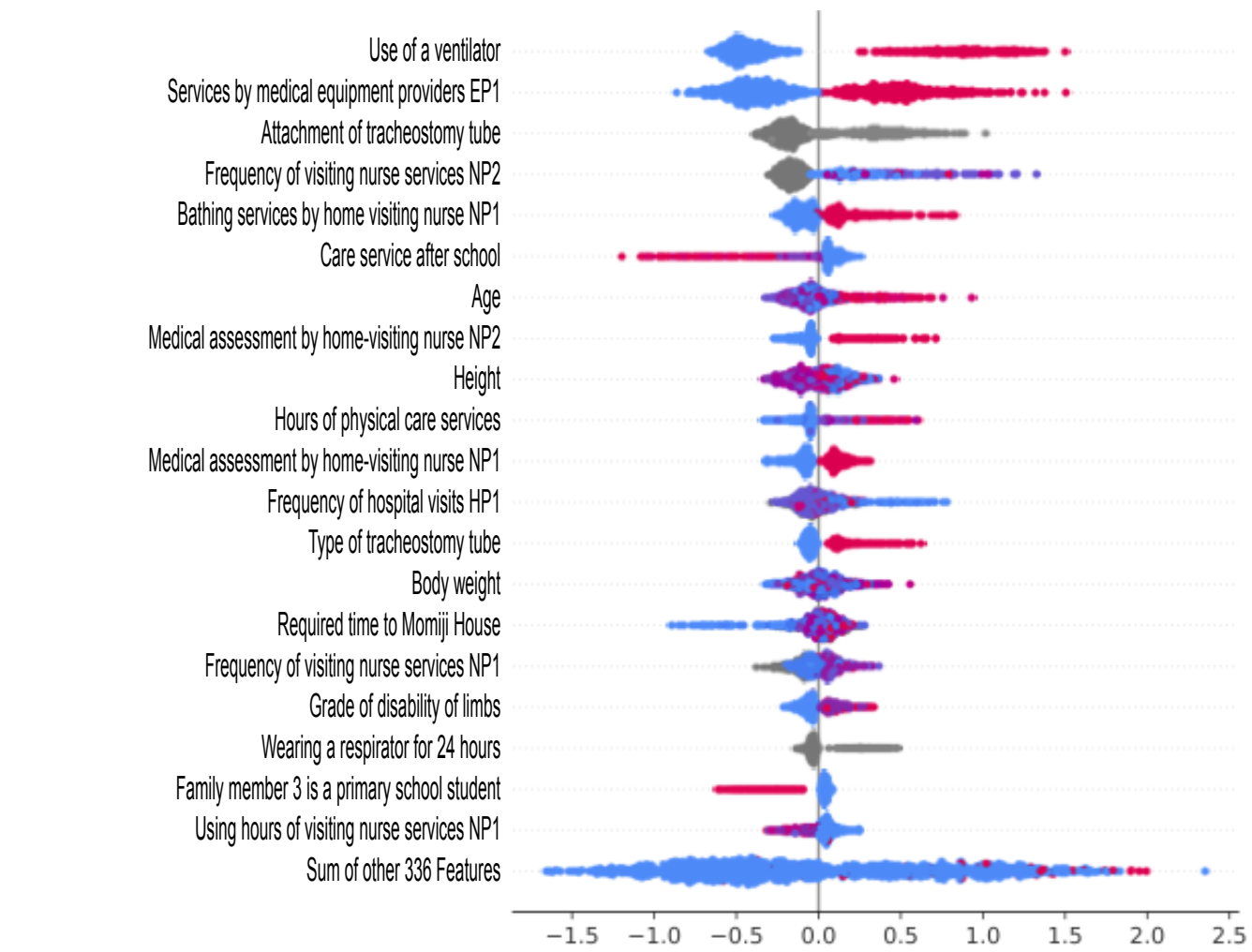

Model #10

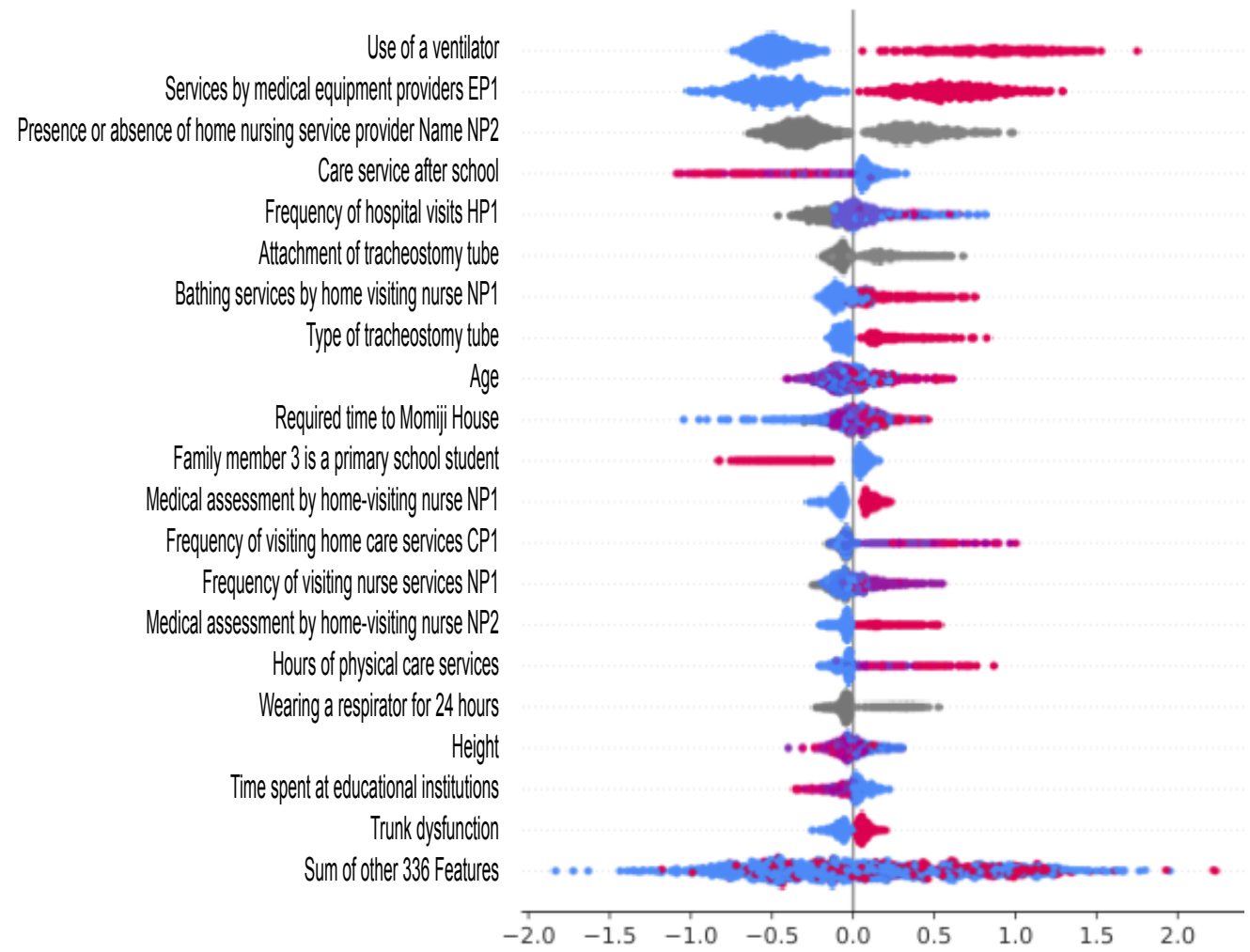

Figure 3

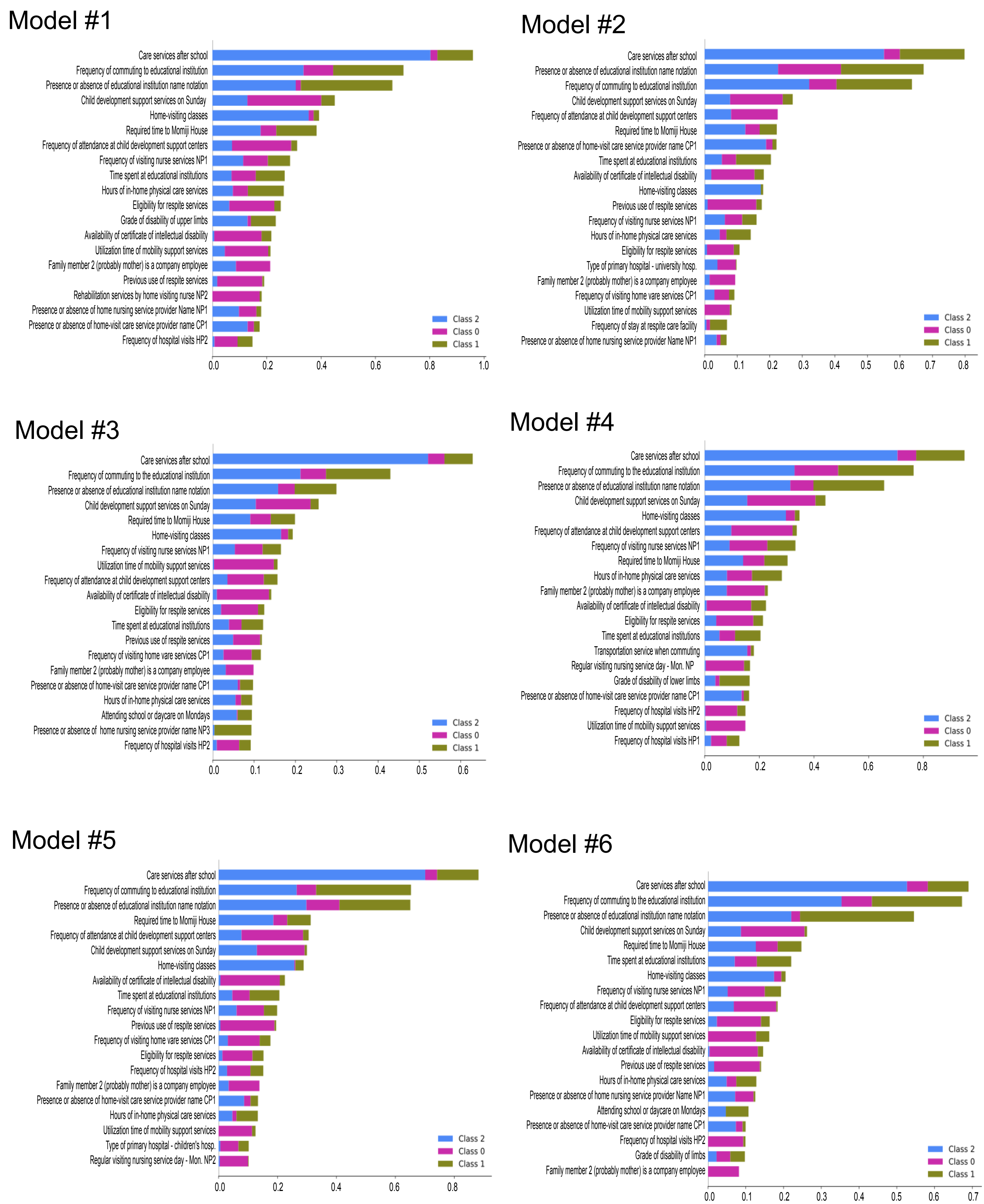

Model #1
Care services after school
Frequency of commuting to educational institution
Presence or absence of educational institution name notation
Child development support services on Sunday
Home-visiting classes
Required time to Momiji House
Frequency of attendance at child development support centers
Frequency of visiting nurse services NP1
Time spent at educational institutions
Hours of in-home physical care services
Eligibility for respite services
Grade of disability of upper limbs
Availability of certificate of intellectual disability
Utilization time of mobility support services
Family member 2 (probably mother) is a company employee
Previous use of respite services
Rehabilitation services by home visiting nurse NP2
Presence or absence of home nursing service provider Name NP1
Presence or absence of home-visit care service provider name CP1
Frequency of hospital visits HP2
Class 2
Class 0
Class 1
0.0
0.2
0.4
0.6
0.8
1.0
Model #2
Care services after school
Presence or absence of educational institution name notation
Frequency of commuting to educational institution
Child development support services on Sunday
Frequency of attendance at child development support centers
Required time to Momiji House
Presence or absence of home-visit care service provider name CP1
Time spent at educational institutions
Availability of certificate of intellectual disability
Home-visiting classes
Previous use of respite services
Frequency of visiting nurse services NP1
Hours of in-home physical care services
Eligibility for respite services
Type of primary hospital - university hosp.
Family member 2 (probably mother) is a company employee
Frequency of visiting home vare services CP1
Utilization time of mobility support services
Frequency of stay at respite care facility
Presence or absence of home nursing service provider Name NP1
Class 2
Class 0
Class 1
0.0
0.1
0.2
0.3
0.4
0.5
0.6
0.7
0.8
Model #3
Care services after school
Frequency of commuting to the educational institution
Presence or absence of educational institution name notation
Child development support services on Sunday
Required time to Momiji House
Home-visiting classes
Frequency of visiting nurse services NP1
Utilization time of mobility support services
Frequency of attendance at child development support centers
Availability of certificate of intellectual disability
Eligibility for respite services
Time spent at educational institutions
Previous use of respite services
Frequency of visiting home vare services CP1
Family member 2 (probably mother) is a company employee
Presence or absence of home-visit care service provider name CP1
Hours of in-home physical care services
Attending school or daycare on Mondays
Presence or absence of home nursing service provider name NP3
Frequency of hospital visits HP2
Class 2
Class 0
Class 1
0.0
0.1
0.2
0.3
0.4
0.5
0.6
Model #4
Care services after school
Frequency of commuting to the educational institution
Presence or absence of educational institution name notation
Child development support services on Sunday
Home-visiting classes
Frequency of attendance at child development support centers
Frequency of visiting nurse services NP1
Required time to Momiji House
Hours of in-home physical care services
Family member 2 (probably mother) is a company employee
Availability of certificate of intellectual disability
Eligibility for respite services
Time spent at educational institutions
Transportation service when commuting
Regular visiting nursing service day - Mon. NP
Grade of disability of lower limbs
Presence or absence of home-visit care service provider name CP1
Frequency of hospital visits HP2
Utilization time of mobility support services
Frequency of hospital visits HP1
Class 2
Class 0
Class 1
0.0
0.2
0.4
0.6
0.8
Model #5
Care services after school
Frequency of commuting to educational institution
Presence or absence of educational institution name notation
Required time to Momiji House
Frequency of attendance at child development support centers
Child development support services on Sunday
Home-visiting classes
Availability of certificate of intellectual disability
Time spent at educational institutions
Frequency of visiting nurse services NP1
Previous use of respite services
Frequency of visiting home vare services CP1
Eligibility for respite services
Frequency of hospital visits HP2
Family member 2 (probably mother) is a company employee
Presence or absence of home-visit care service provider name CP1
Hours of in-home physical care services
Utilization time of mobility support services
Type of primary hospital - children's hosp.
Regular visiting nursing service day - Mon. NP2
Class 2
Class 0
Class 1
0.0
0.2
0.4
0.6
0.8
Model #6
Care services after school
Frequency of commuting to the educational institution
Presence or absence of educational institution name notation
Child development support services on Sunday
Required time to Momiji House
Time spent at educational institutions
Home-visiting classes
Frequency of visiting nurse services NP1
Frequency of attendance at child development support centers
Eligibility for respite services
Utilization time of mobility support services
Availability of certificate of intellectual disability
Previous use of respite services
Hours of in-home physical care services
Presence or absence of home nursing service provider Name NP1
Attending school or daycare on Mondays
Presence or absence of home-visit care service provider name CP1
Frequency of hospital visits HP2
Grade of disability of limbs
Family member 2 (probably mother) is a company employee
Class 2
Class 0
Class 1
0.0
0.1
0.2
0.3
0.4
0.5
0.6
0.7

Model #7

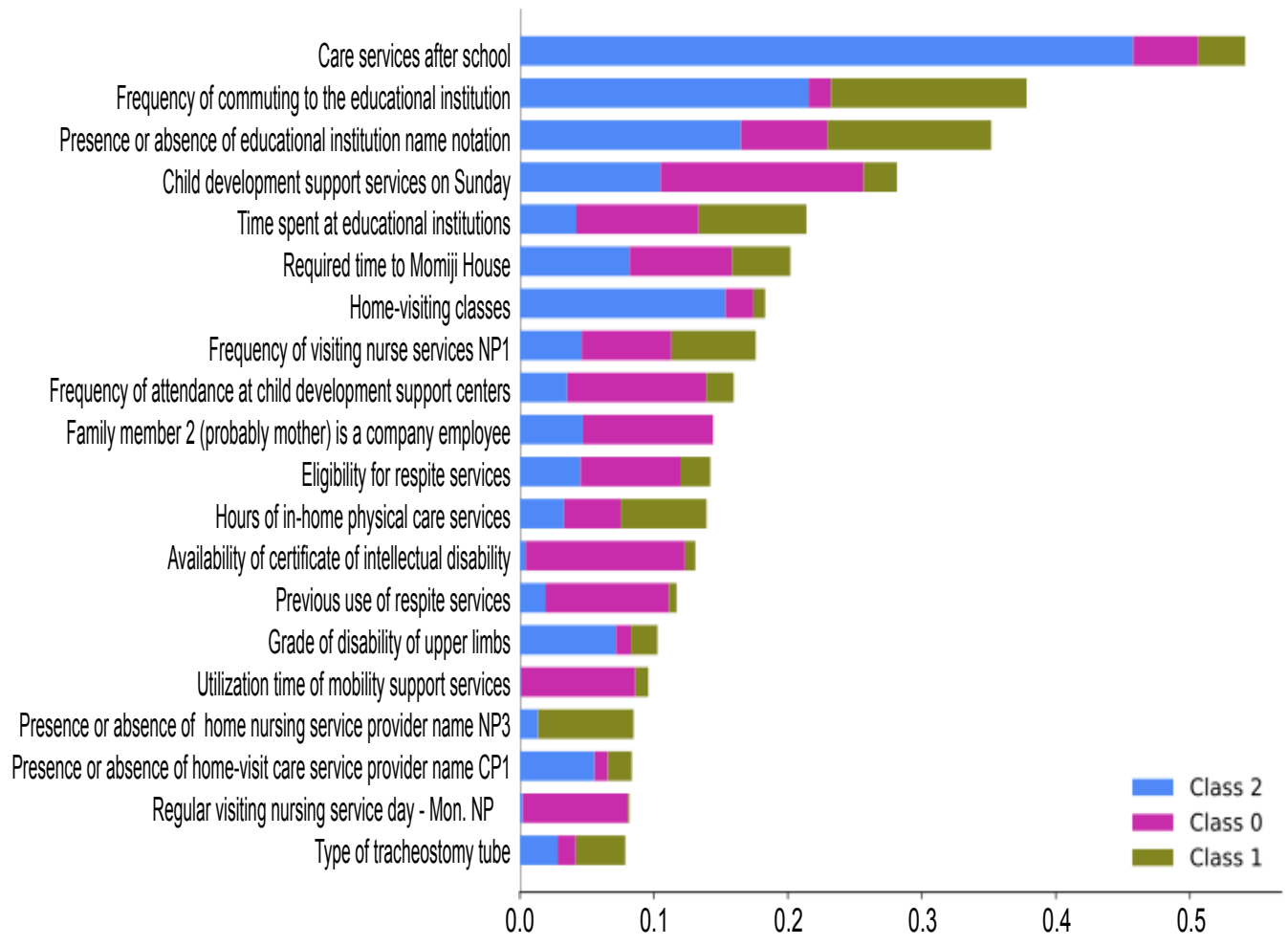

Model #8

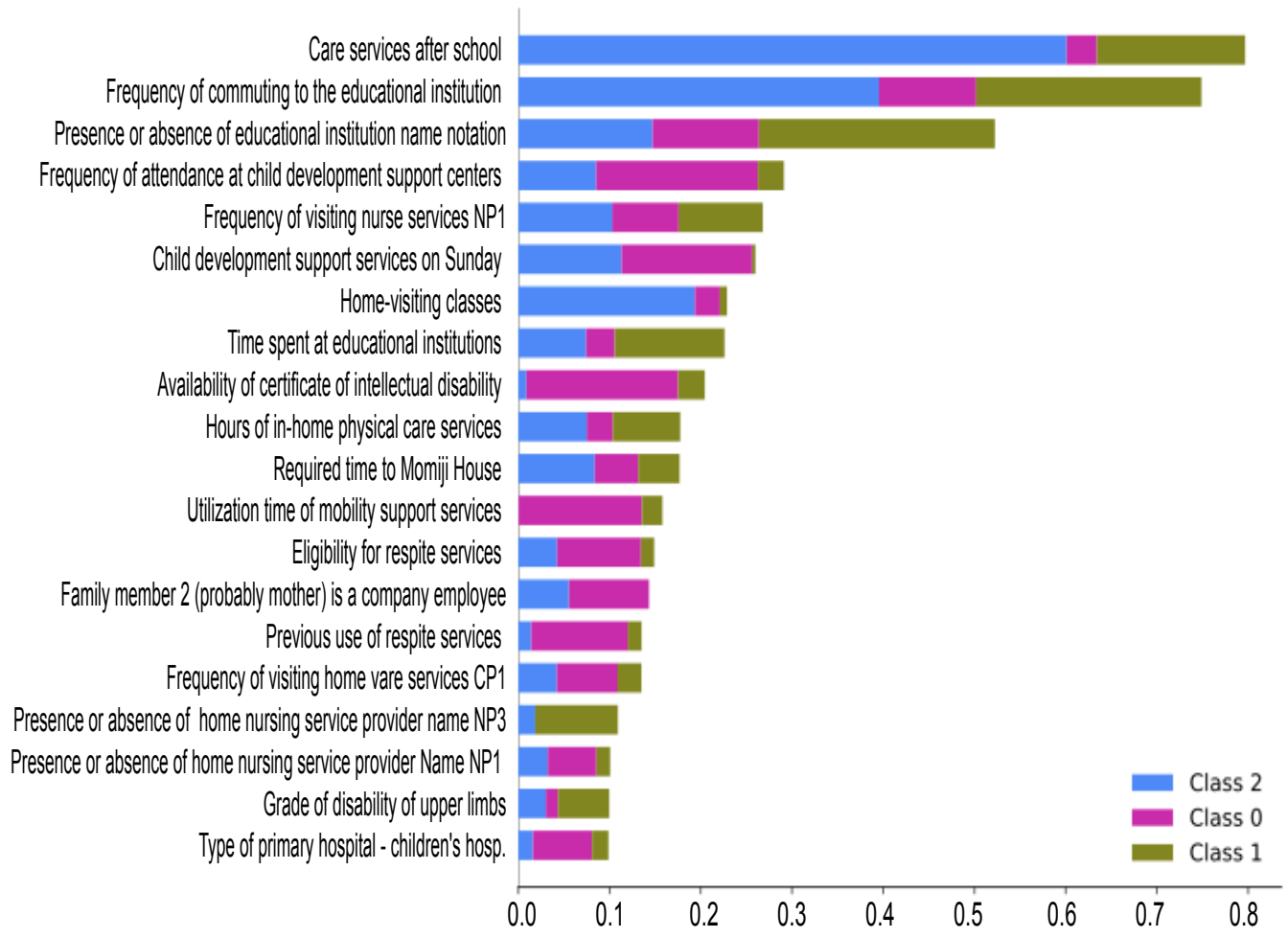

Model #9

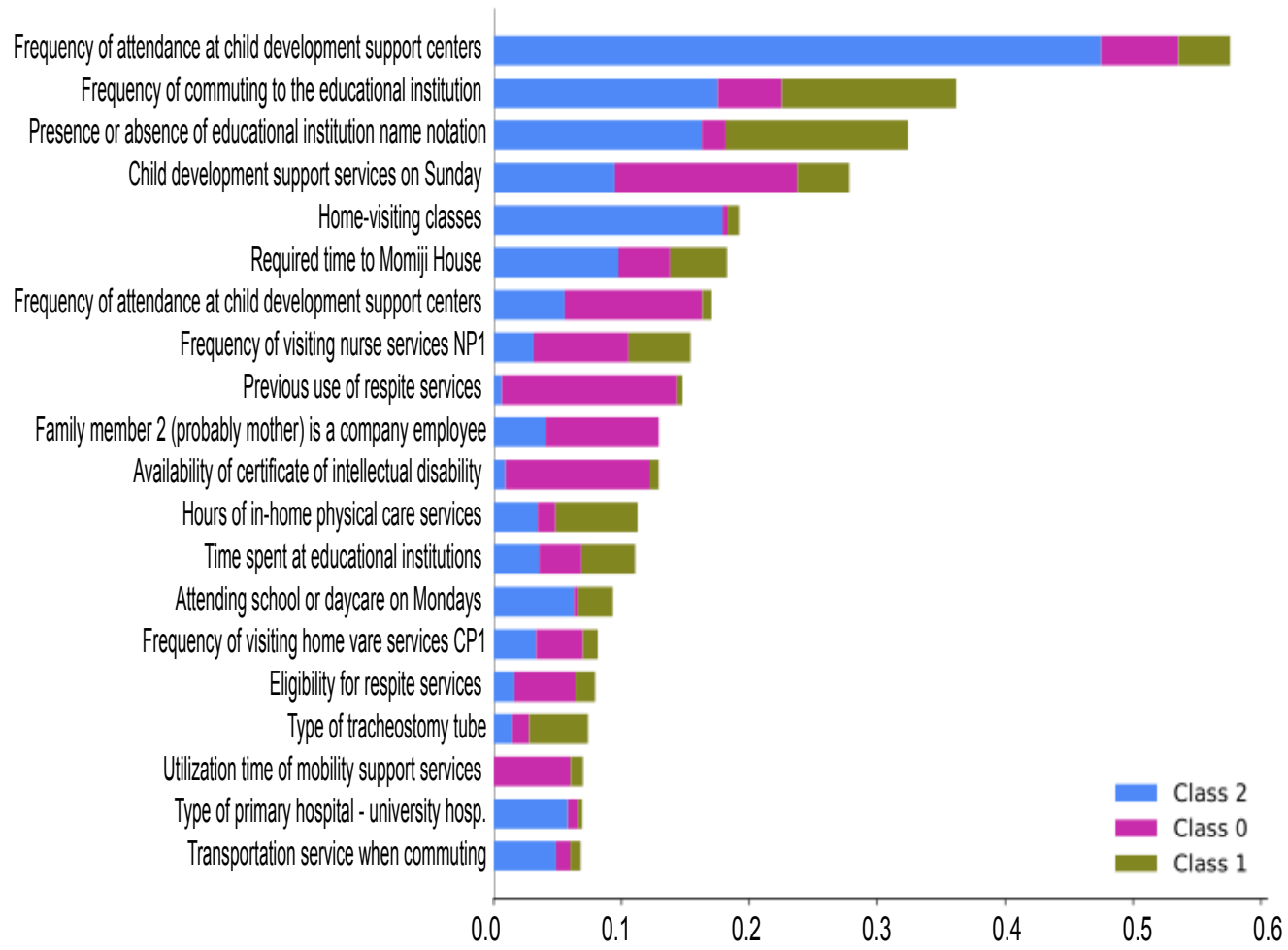

Model #10

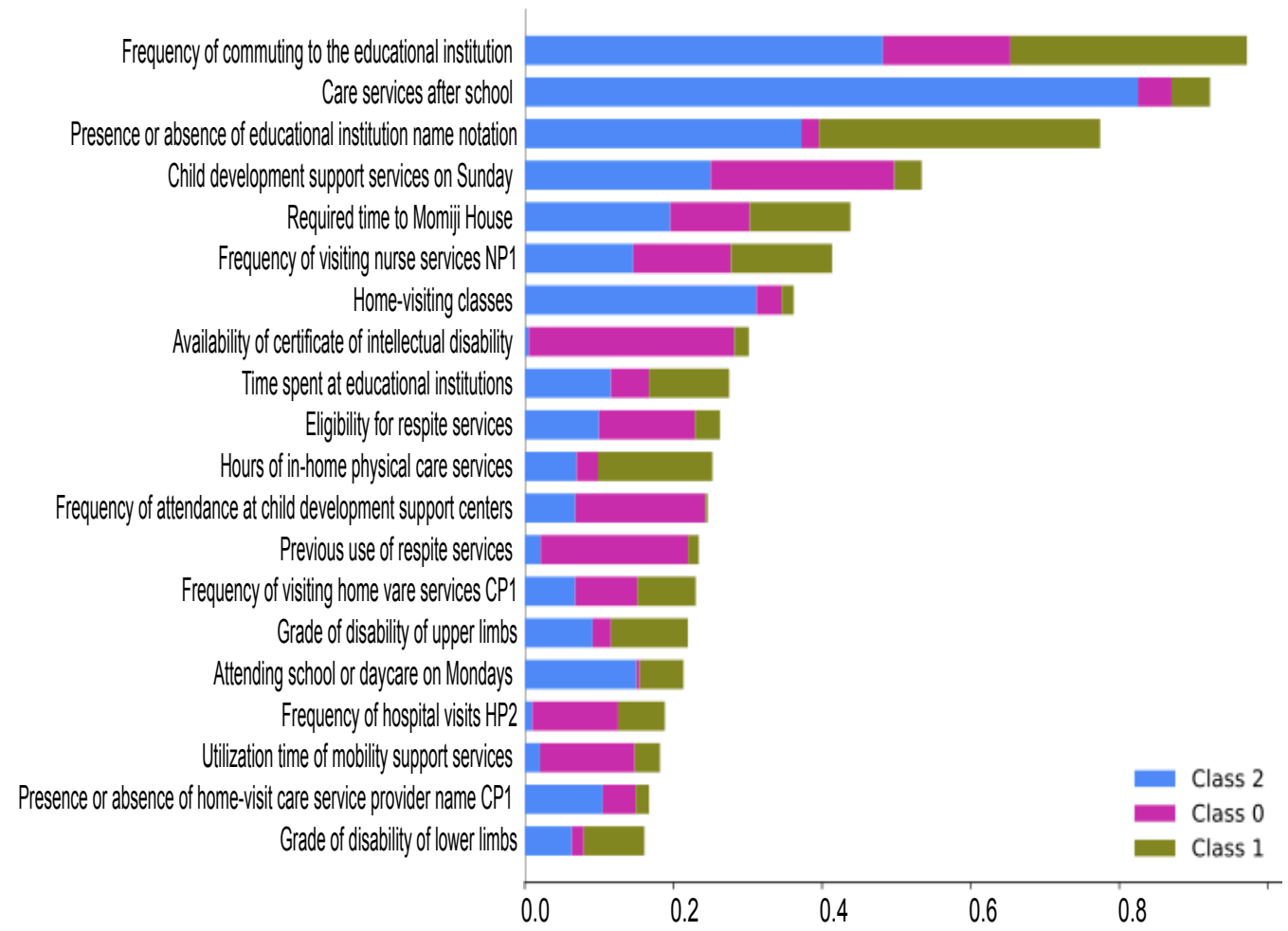

Supplement: Supplementary file 1 [file 12911_2026_3691_MOESM2_ESM.pdf]
